# Structure and co-occurrence patterns of bacterial communities associated with white faeces disease outbreaks in Pacific white-leg shrimp *Penaeus vannamei* aquaculture

**DOI:** 10.1038/s41598-020-68891-6

**Published:** 2020-07-20

**Authors:** Yustian Rovi Alfiansah, Sonja Peters, Jens Harder, Christiane Hassenrück, Astrid Gärdes

**Affiliations:** 1 Leibniz Centre for Tropical Marine Research (ZMT), 28359 Bremen, Germany; 2 Research Center for Oceanography (RCO-LIPI), Jakarta, 14430 Indonesia; 3 Center for Aquaculture Research (ZAF), Alfred Wegener Institute (AWI), 27570 Bremerhaven, Germany; 4 Department of Molecular Ecology, Max Planck Institute for Marine Microbiology (MPI-MM), 28359 Bremen, Germany; 5 Division Biosciences/Polar Biological Oceanography, Alfred Wegener Institute (AWI), 27570 Bremerhaven, Germany; 6 Hochschule (HS) Bremerhaven, 27568 Bremerhaven, Germany

**Keywords:** Ecology, Microbiology, Molecular biology, Environmental sciences

## Abstract

Bacterial diseases cause production failures in shrimp aquacultures. To understand environmental conditions and bacterial community dynamics contributing to white faeces disease (WFD) events, we analysed water quality and compared bacterial communities in water as well as in intestines and faeces of healthy and diseased shrimps, respectively, via 16S rRNA gene sequencing and qPCR of transmembrane regulatory protein (*toxR*), thermolabile haemolysin (*tlh*), and thermostable direct haemolysin genes of pathogenic *Vibrio parahaemolyticus* as a proxy for virulence. WFD occurred when pH decreased to 7.71–7.84, and *Alteromonas*, *Pseudoalteromonas* and *Vibrio* dominated the aquatic bacterial communities. The disease severity further correlated with increased proportions of *Alteromonas*, *Photobacterium, Pseudoalteromonas* and *Vibrio* in shrimp faeces. These opportunistic pathogenic bacteria constituted up to 60% and 80% of the sequences in samples from the early and advances stages of the disease outbreak, respectively, and exhibited a high degree of co-occurrence. Furthermore, *toxR* and *tlh* were detected in water at the disease event only. Notably, bacterial community resilience in water occurred when pH was adjusted to 8. Then WFD ceased without a mortality event. In conclusion, pH was a reliable indicator of the WFD outbreak risk. Dissolved oxygen and compositions of water and intestinal bacteria may also serve as indicators for better prevention of WFD events.

## Introduction

Bacterial diseases are a major problem for *Penaeus vannamei* pond aquaculture in Asia and Latin America. They have been causing severe annual economic losses reaching approximately USD 1 billion over last decade^1,2^. Among reported bacterial diseases, acute hepatopancreatic necrosis disease (AHPND) and white faeces disease (WFD) are the most infectious and lethal ones^3,4^. The latter has frequently been occurring in Asian shrimp aquaculture since 2009^3,5,6^, which reduced shrimp survival to 20–30%^6^.

WFD events are characterized by the presence of white faecal strings which float in the rearing water^3,6^. They usually occur after approximately 50 days of culture^6^, resulting in retarded shrimp growth, unprofitable harvests, and even mass mortality^7^. Loss of microvilli and subsequent lysis in hepatopancreas and midgut associated with WFD indicate a pathological process in shrimp’s gut^6^. *Enterocytozoon hepatopenaei* (EHP), vermiform bodies resembling protozoan gregarines, and certain culturable *Vibrio* species, such as *V. parahaemolyticus*, *V. fluvialis*, *V. mimicus*, *V. alginolyticus*, and *Vibrio sp.* were reported as potential causative agents of the disease^3,6,8,9^. Furthermore, deteriorated water quality with oxygen concentrations below 3.0 mg L^−1^ and alkalinity below 80 ppm caused peak mortality rates during WFD outbreaks^10^. However, the aetiology of the WFD in shrimp pond farming remains inconclusive^6,8,11^. For instance, EHP-infected shrimps did not always produce white faeces^11^, while *Vibrio* such as *V. alginolyticus* was also reported to be probiotic for *P. vannamei*^12,13^.

Often antibiotics and viable beneficial bacterial cells (probiotics) are applied in shrimp farming to enhance shrimp growth and to avoid disease outbreaks^14–17^, without success. Common assessment of culturable heterotrophic bacterial numbers, as well as *Vibrio* counts on thiosulfate-citrate-bile salts-sucrose (TCBS) medium, failed to predict the pathogenic event. Surprisingly, WFD still happened even though culturable *Vibrio* counts were threefold lower than culturable heterotrophic bacteria (pers. comm. with shrimp pond owners). In addition, the viable plate count method, which selects certain pathogenic bacteria^18^, was shown to be inadequate to identify the bacterial population^19^, which may be associated with the disease. Thus, these practices have been unsuccessful to predict WFD in *P*. *vannamei* aquaculture.

The composition of intestinal bacteria has a strong influence on shrimp health^17,20,21^. For instance, WFD can be initiated in healthy shrimps by transplantation of intestinal microbiota of diseased shrimp^22^. Bacterial community composition (BCC) in shrimp intestines may dynamically change following shrimp development^23^ and diets^16,24,25^. In addition, shrimp habitat, i.e. the water column and the underlying sediment, may affect intestinal bacteria (IB) with those of wild shrimps differing from those of domesticated/cultured shrimps^26,27^. Yet, there is little information about the interaction between rearing water parameters, IB, faecal string bacteria (FSB) and BCC in pond waters before, during, and after disease outbreaks. Also, investigation of bacterial communities in pond water (WB) during WFD events is still neglected. Thus, more extensive information of bacterial community dynamics including pathogenic bacteria in pond water and in association with the shrimps at disease and non-disease stages is needed to understand and prevent WFD, and to treat diseased shrimps. We further propose that sudden changes of water quality will affect firstly bacterial communities in pond water (water bacteria/WB) and subsequently shrimp physiology and their IB.

In the water column, bacteria occur free-living (FL) or particle-associated (PA), or alternating between these different lifestyles^28^. As opportunistic pathogenic microorganisms are known to favour a particle-associated lifestyle, particles, especially larger aggregates, may constitute pathogen hotspots^29–31^, while simultaneously serving as an alternative feed for shrimps and fish^32,33^. During WFD events, faecal strings, containing among others opportunistic pathogenic bacteria, disintegrate in the rearing water and faecal bacteria are released. They may then enrich the WB^34^ resulting in a high load of potential opportunistic pathogenic bacteria predominantly in the particulate fraction. We therefore hypothesize that the outbreak of the disease can be facilitated by consumption of contaminated aggregates. Thus, a separate monitoring of FL and PA bacteria is necessary to predict disease transfer among shrimps in a closed shrimp aquaculture system.

To address the research needs and hypotheses outlined above, we provide a comprehensive overview of the bacterial dynamics and the water quality in shrimp ponds over the course of a WFD event, by (i) investigating water quality parameters, (ii) elucidating the BCC in rearing water (WB), separated into FL and PA bacteria, in the intestines of healthy *L. vannamei* (IB), and in white faecal strings (FSB), (iii) quantifying pathogenic *Vibrio* by their virulence gene copy numbers, and (iv) analysing bacterial co-occurrence patterns in healthy and diseased shrimps. We conducted 16S rRNA gene amplicon sequencing and virulence factor gene quantification via qPCR of the transmembrane regulatory protein (*toxR*), thermolabile haemolysin (*tlh*), and thermostable direct haemolysin (*tdh*) of pathogenic *V*. *parahaemolyticus*. Furthermore, we discriminated co-occurring bacterial sub-population characteristics for healthy and diseased shrimps.

## Results

The WFD events investigated in this study occurred in shrimp ponds, whose water parameters and WB during the full rearing cycle at non-disease events have been reported elsewhere^35^. The WFD events occurred in ponds with moderate (P2) and high stocking densities (P3 and P4) at the 52nd, 63th and 67th day of rearing, respectively, suggesting that the disease may happen regardless of the density of the reared shrimps. The WFD event coincided with a sudden change in pond water parameters, a shift in the WB, and stress in the cultured shrimps, indicated by a decrease of appetite 2–3 days prior to the onset of the disease.

### Biogeochemical characteristics of the shrimp pond water

Ponds with infected shrimps were characterized by lower pH (7.71–7.84), dissolved oxygen/DO (5.57–5.98 mg mL^−1^), higher turbidity (38.0–41.7 NTU) and contained more culturable non-sucrose fermenting presumptive *Vibrio* colonies (4,000–4,700 CFU mL^−1^). In contrast, the pond with healthy shrimps (P1) had higher pH (> 8), DO (> 6 mg mL^−1^), lower turbidity (< 30 NTU), and fewer CFU-counts of non-sucrose fermenting presumptive pathogenic *Vibrio* colonies (0–400 CFU mL^−1^; Table 1). Considering the low pH during the WFD events, the shrimp pond owners added limestone at night after they observed the first symptoms of the disease. This treatment was performed until the symptoms of the disease disappeared. They added approximately 0.4–1.5 tons per pond (approximated water volume 3,500–3,700 m^3^) for 3 days. This treatment affected the water quality, particularly the pH value, which increased to above 8, while numbers of non-sucrose fermenting presumptive pathogenic *Vibrio* decreased 3 to sixfold after the WFD outbreaks (Table 1).

**Table 1:** Biogeochemical parameters of the pond with healthy shrimps (P1) and the ponds which experienced white faeces disease (P2, P3, P4) before, during (in bold) and after disease events.

Parameters	Pond 1			Pond 2			Pond 3			Pond 4		
Sampling time (day)	50	60	70	50	**53**	60	60	**63**	70	60	**67**	70
pH	8.12	8.40	8.21	8.18	**7.79**	7.93	8.02	**7.84**	7.96	7.74	**7.71**	8.17
Temperature (°C)	30.32	29.91	30.05	31.14	**30.94**	30.94	29.56	**30.61**	30.87	30.29	**30.23**	30.57
DO (mg L^−1^)	6.10	6.10	6.20	5.60	**5.57**	5.80	6.32	**5.98**	6.02	5.93	**5.68**	6.58
Turbidity (NTU)	19.60	25.60	18.50	19.10	**41.70**	25.60	25.70	**38.50**	38.60	30.70	**38.00**	49.90
Chl a (mg L^−1^)	88.66	21.19	39.60	28.96	**45.76**	10.22	70.28	**69.76**	69.79	31.64	**45.97**	94.90
Salinity (PSU)	35.53	34.47	33.36	32.92	**34.73**	34.02	36.12	**34.17**	34.09	35.64	**34.02**	33.27
SPM (mg L^−1^)	181.17	194.82	151.88	161.49	**184.34**	168.74	193.03	**196.38**	186.52	174.94	**183.47**	175.60
**TPPV (CFU mL**^**−1**^**)**												
suc(−)	0	0	400	400	**4,000**	600	300	**4,700**	700	300	**4,000**	1,100
suc( +)	7,800	6,700	3,000	4,100	**1,700**	6,400	3,700	**1,700**	9,700	4,400	**2000**	8,800
**Inorganic nutrients (mg L**^**−1**^**)**												
Phosphate (PO_4_^3−^)	0.063	0.886	0.086	0.914	**0.795**	0.095	0.245	**0.793**	0.524	0.031	**0.770**	0.333
Nitrite (NO_2_^−^)	0.001	0.219	0.001	0.019	**0.199**	0.002	0.001	**0.217**	0.006	0.002	**0.205**	0.005
Nitrate (NO_3_^−^)	0.029	0.076	0.006	0.046	**0.061**	0.061	0.011	**0.078**	0.003	0.003	**0.087**	0.842
Ammonium (NH_4_^+^)	0.662	1.017	0.186	0.482	**1.088**	0.088	0.373	**1.060**	0.650	0.062	**1.041**	0.236
Reactive silicate	0.185	0.643	2.775	0.394	**0.554**	1.554	0.859	**0.803**	0.494	0.297	**0.566**	0.197

*DO* dissolved oxygen, *SPM* suspended particulate matter, *Chl a* chlorophyll a, *TPPV* total culturable presumptive pathogenic *Vibrio*, *suc(−)* non-sucrose fermenting colonies (green colonies), *suc(+)* sucrose fermenting colonies (yellow colonies).

**Figure 1:**
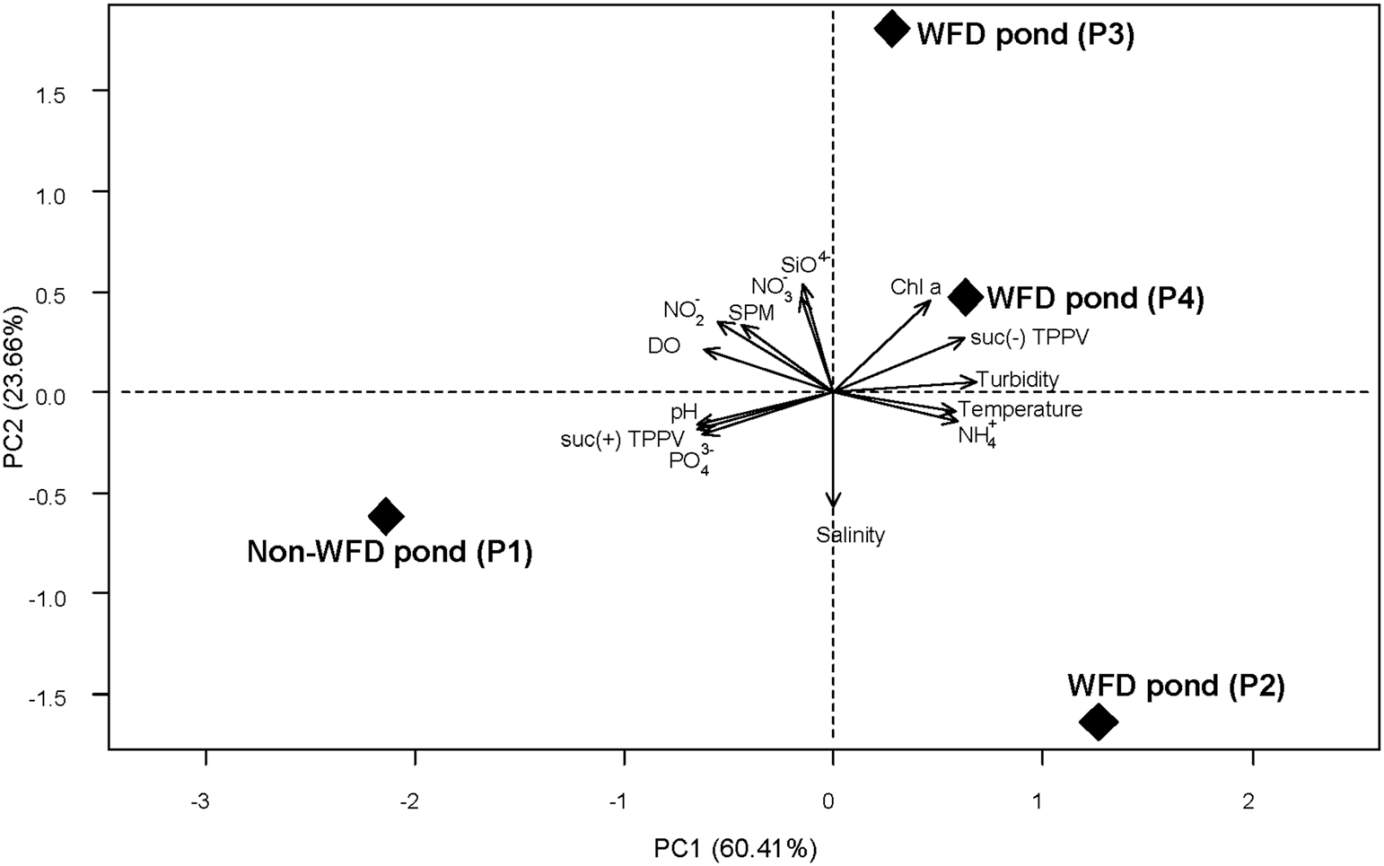
Principal component analysis (PCA) of observed environmental parameters. Point labels indicate the pond without white faeces disease (non-WFD pond P1) and ponds with disease events (P2, P3, and P4). Water parameters presented in the PCA were taken at day 60, 53, 63, and 67 for P1, P2, P3, and P4, respectively. SPM: suspended particulate matter; suc(+) TPPV: sucrose-fermenting colonies of total presumptive pathogenic *Vibrio* (yellow colonies of presumptive pathogenic *Vibrio*); suc(−) TPPV: non sucrose-fermenting colonies of presumptive pathogenic *Vibrio* (green colonies); *DO* dissolved oxygen; *Chl a* chlorophyll a concentration; NO_2_^-^: nitrite; NO_3_^−^: nitrate; NH_4_^+^: ammonium; r-Silicate: reactive silicate; PO_4_^3−^: phosphate.

Environmental parameters in P1 at day 60th and in P2, P3, and P4 at WFD outbreaks were plotted in a PCA to characterize the shrimp ponds (Fig. 1). The ponds with healthy and diseased shrimps were separated along PC1, which accounted for 60% of the variation in the data, and was determined mostly by the abundances of culturable presumptive pathogenic *Vibrio*, ammonium and phosphate concentrations, pH, temperature, and turbidity. Nitrate and reactive silicate concentrations, and salinity were among the water parameters which contributed most to PC2.

### Bacterial community composition (BCC)

A total of 80 samples from pond water, shrimp intestines, white faecal strings, presumptive pathogenic *Vibrio* strains and commercial probiotic were sequenced, resulting in 3,917,111 high quality sequences ranging from 7,892 to 200,098, with a mean of 48,963 sequences per sample. After merging the operational taxonomic unit (OTU) profiles of the technical replicates that were collected for the FL and the PA fractions in P2, P3, and P4 at WFD events, the sequencing data set for bacterial community analyses consisted of 70 samples with on average 58,336 sequences per sample. Cluster analysis and non-metric multidimensional scaling (NMDS) of these samples showed highly heterogeneous bacterial communities, which were grouped in seven clusters at a Bray–Curtis dissimilarity threshold of 0.95. Within each cluster average Bray–Curtis dissimilarities ranged from 0.51 to 0.72. The WB in FL and PA fractions at non-disease events clustered in two groups, which were distinct from WB at disease events. Sequences generated from the bacterial strains grown on the TCBS agar from P1 were exclusively affiliated with the genus *Vibrio*. They clustered together with IB from P1, which were likewise dominated by *Vibrio* **(**Supplementary Information Fig. 1).

**Figure 2:**
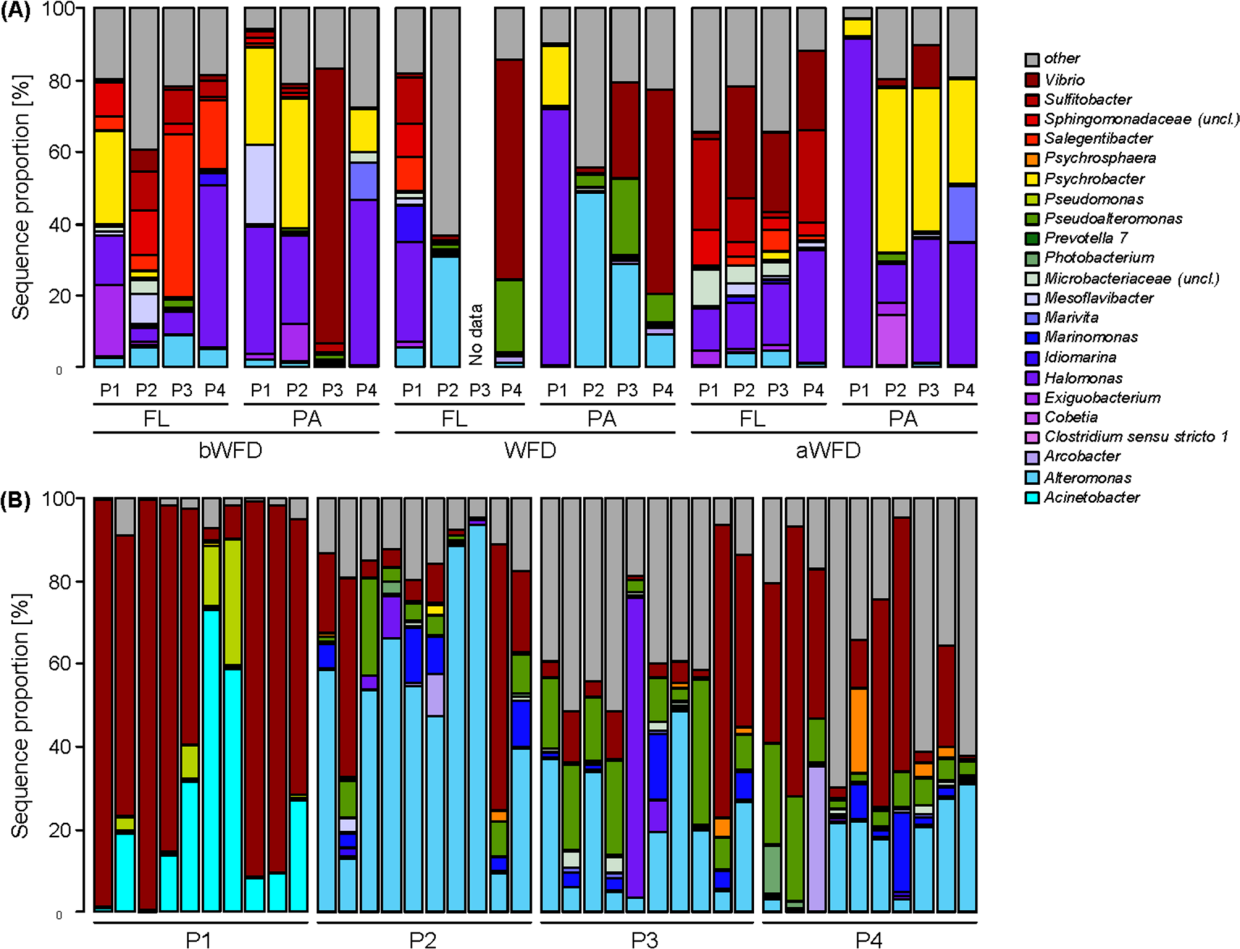
Contribution of the most dominant bacterial genera in pond water communities (**A**) and shrimp intestines and faeces (**B**). Samples were collected from a pond with healthy shrimps (P1) and ponds with diseased shrimps (P2, P3, and P4). Intestine (P1) for intestinal bacteria (IB) and faecal strings (P2, P3, P4) for faecal string bacteria (FSB) were sampled at rearing day 60, 53, 63, and 67 for P1, P2, P3, and P4, respectively. *FL* free-living fraction, *PA* particle-associated fraction, *bWFD* before WFD event, *WFD* during WFD event, *aWFD* after WFD event.

Despite the high overall heterogeneity, bacterial communities in pond water (WB) in P1 showed a similar composition of dominant bacterial taxa at all investigated sampling times. Based on 16S rRNA sequencing, the WB in P1 was predominantly comprised of the bacterial taxa *Salegentibacter* (*Bacteroidia*), *Exiguobacterium* (*Bacilli*), and *Halomonas* and *Psychrobacter* (*Gammaproteobacteria*). These taxa were also found in the WB of the FL and the PA fractions of P2, P3, and P4 before and after the disease event (Fig. 2A). During the disease event, the WB of P2, P3, and P4 were altered with *Mesoflavibacter* (*Bacteroidia*), *Arcobacter* (*Campylobacteria*), and *Alteromonas*, *Marinomonas*, *Photobacterium*, *Pseudoalteromonas* and *Vibrio* (*Gammaproteobacteria*) dominating BCC (Fig. 2A). Those genera exhibited only low proportions in the WB of P1 at all sampling points and in both fractions, with the exception of *Vibrio*.

Dominant members of intestinal bacteria (IB) were *Gammaproteobacteria* of the genera *Acinetobacter*, *Pseudomonas*, and *Vibrio*, while faecal string bacteria (FSB) samples were dominated by *Arcobacter* (*Campylobacteria*) and *Gammaproteobacteria* of the genera *Alteromonas*, *Marinomonas*, *Photobacterium*, *Pseudoalteromonas* and *Vibrio* (Fig. 2B). Interestingly, neither *Acinetobacter* nor *Pseudomonas* affiliated sequences were found in FSB. Conversely, *Alteromonas*, *Marinomonas*, *Photobacterium* and *Pseudoalteromonas* were absent in healthy shrimp intestines. This clear distinction between IB and FSB was further supported by pairwise ANOSIM test, which showed that IB differed from the FSB of P2, P3, and P4, while FSB among ponds with infected shrimps were more similar (Table 2). Especially, in the faecal string (FS) samples from P2, *Alteromonas* made up more than 50% of all sequences in seven out of ten samples, while in the remaining three samples, *Alteromonas* still constituted up to 40%.

**Table 2:** Pairwise analysis of similarity (ANOSIM) and Bray–Curtis dissimilarity values comparing bacterial community compositions (BCC) in the intestine of healthy shrimps (IB) and that of white faecal strings (FSB).

BCC	MI P1	MFS P2	MFS P3	MFS P4
IB P1	*0.63*	0.92	0.95	0.93
FSB P2	(**0.80**, 0.002)	*0.65*	0.75	0.80
FSB P3	(**0.87**, 0.002)	(0.22, 0.006)	*0.70*	0.73
FSB P4	(**0.75**, 0.002)	(0.24, 0.002)	(-0.01, 0.523)	*0.75*

*P1* pond with healthy shrimps; *P2, P3 and P4* ponds with diseased shrimps. Lower triangle: ANOSIM R values and Benjamini–Hochberg adjusted p values (in parenthesis). Values written in bold indicate strongly separated communities. Diagonal: average Bray–Curtis dissimilarity within pond (in italics). Upper triangle: average Bray–Curtis dissimilarity between ponds (underlined). Sample number per pond: N = 10.

**Figure 3:**
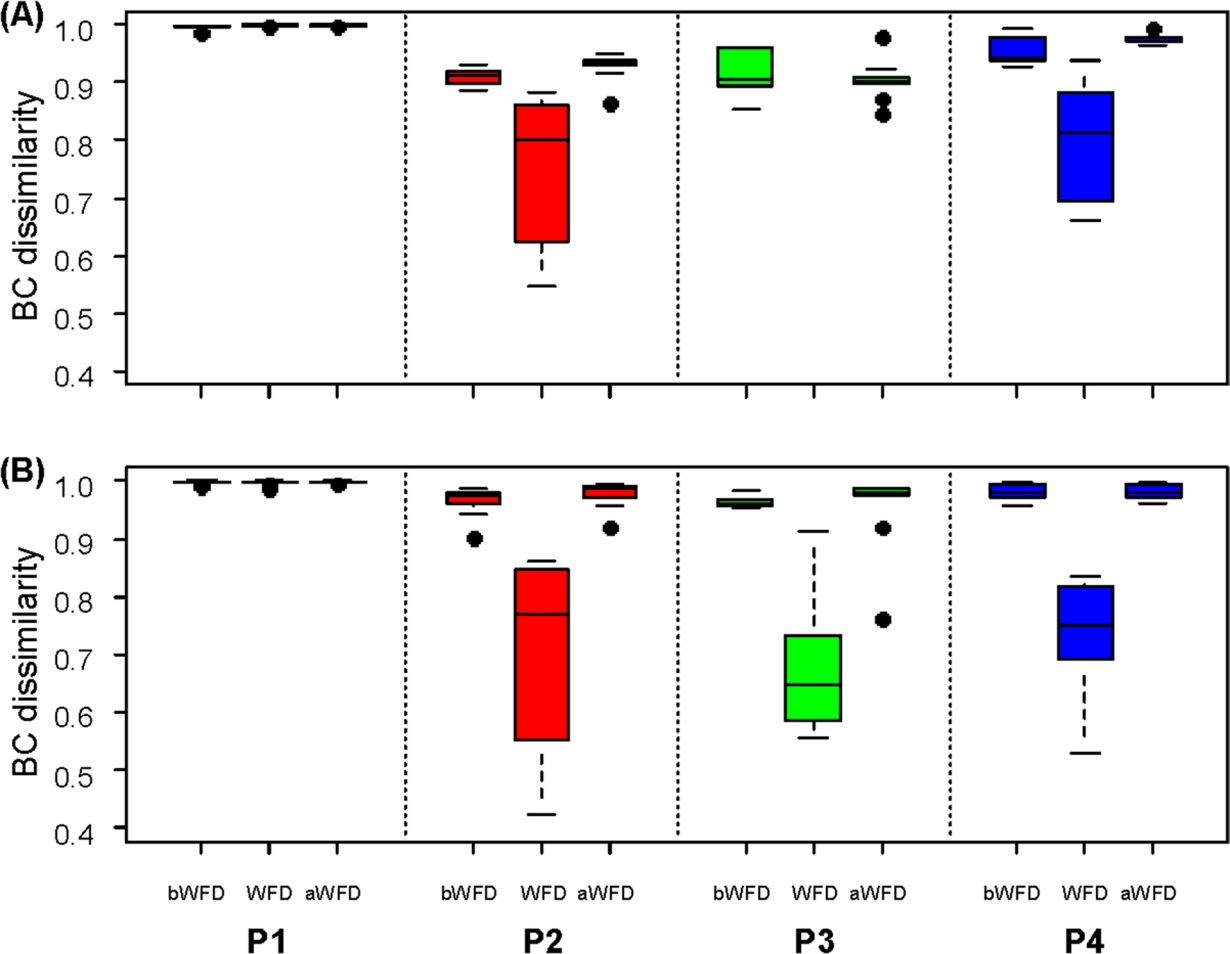
Bray–Curtis dissimilarity values of WB in the free-living (**A**) and particle-associated (**B**) fractions compared to the intestinal (IB) or faecal string bacteria (FSB) for samples from P1 and P2-P4, respectively. bWFD: before WFD event, WFD: during WFD event, aWFD: after WFD event. P1: pond with healthy shrimps; P2, P3 and P4: ponds with diseased shrimps.

WB of the FL and PA fractions from ponds with infected shrimps at non-disease events were highly dissimilar to FSB. However, during disease events, FSB and WB shared similar bacterial community compositions as indicated by consistently decreased Bray–Curtis dissimilarity values in all diseased ponds (Fig. 3; Supplementary Table 1). In contrast, in the pond with healthy shrimps, IB and WB were highly dissimilar at all sampling times (Fig. 3).

### Virulence gene detection and quantification

Our primer pairs detected *toxR*, *tlh*, and *tdh* genes from the positive control *V. parahaemolyticus* DSM 10,143 with a limit of quantification of 26 cells mL^−1^ (Supplementary Tables 2 and 3). We targeted these three genes in WB, IB, and FSB samples, but only two virulence genes (*toxR* and *tlh)* could be detected and quantified (Table 3). Concentrations (copy numbers) of the *toxR* and *tlh* gene in intestines and FS did not differ from each other (Table 3). They varied in a range from 3.7 to 4.5 and 3.5 to 4.3 log gene copies, which were equal to 4,926 to 33,665 and 3,140 to 19,907 gene copies per mL volume of faecal string or intestine for *toxR* and *tlh*, respectively.

**Table 3:** Concentration of *toxR* and *tlh* genes in shrimp (intestines of healthy shrimps and faecal strings of diseased shrimps) and pond water samples.

Sample	Pond	N	% Q (Nq/N)	Min–Max Q level		Mean Q ± SD		ANOVA		Q Unit (Log copies)
			*toxR*, *tlh*	*toxR*	*tlh*	*toxR*	*tlh*	*toxR*	*tlh*	
**Shrimp**										
Intestine	P1	10	90 (9/10)	1.2–6.9	0.9–6.9	4.5 ± 1.8	3.9 ± 2.5	df: 3, 36 F-value: 0.71 *p*: 0.55	df: 3, 36 F-value: 0.14 *p*: 0.93	per mL volume of intestine
Fecal string	P2	10	90 (9/10)	1.5–5.0	0.9–5.5	3.7 ± 1.0	3.7 ± 1.2			per mL volume of faecal string
Fecal string	P3	10	100 (10/10)	2.1–6.6	1.6–6.6	3.7 ± 1.4	3.5 ± 1.5			
Fecal string	P4	10	90 (9/10)	1.6–6.4	2.1–6.5	3.9 ± 1.5	4.3 ± 1.6			
**Water**										
PA	P1	3	0	< LoQ	< LoQ	< LoQ	< LoQ	df: 5, 12 F-value: 49.05 *p*: < 0.001	df: 5, 12 F-value: 126.08 *p*: < 0.001	per L of pond water
	P2	3	100 (3/3)	13.9–15.9	11.7–13.8	14.9 ± 1.3^**a**^	13.0 ± 1.2^**a**^			
	P3	3	100 (3/3)	3.4–6.7	6.1–7.0	4.6 ± 1.9^**bc**^	6.7 ± 0.5^**b**^			
	P4	3	100 (3/3)	6.4–6.8	5.8–6.4	6.7 ± 0.2^**b**^	6.0 ± 0.3^**b**^			
FL	P1	3	0	< LoQ	< LoQ	< LoQ	< LoQ			
	P2	3	100 (3/3)	11.7–14.2	14.2–16.1	13.2 ± 1.3^**a**^	14.9 ± 1.0^**a**^			
	P3	3	100 (3/3)	2.9–4.7	1.5–2.6	5.0 ± 0.8^**bc**^	2.2 ± 0.6^**c**^			
	P4	3	100 (3/3)	4.3–5.9	4.0–5.4	3.8 ± 0.9^**c**^	4.7 ± 0.7^**b**^			

Water samples are separated into free-living (FL) and particle-associated (PA) fractions.

*N* number of samples for intestine and faecal string, and replicates for water samples; *Q* quantified samples; *SD* standard deviation; *PA* particle-associated fraction; *FL* free-living fraction; *LoQ* limit of quantification. Different superscript letters after values in PA and FL fractions of water samples indicate that samples differed significantly according to TukeyHSD post-hoc tests. Copy numbers of *toxR* and *tlh* genes were tested separately.

Concentration of *toxR* and *tlh* genes in the pond water differed significantly between FL and PA fractions (*toxR*: MANOVA, Pillai_2,6_ = 0.623, *p* = 0.05; *tlh*: MANOVA, Pillai_2,6_ = 0.854, *p* < 0.05). The PA and FL fraction from P2 water contained higher *toxR* gene copy numbers (14.9 ± 1.3 and 13.2 ± 1.3 log copies L^−1^, respectively), which differed from the respective fractions of the two other ponds with diseased shrimps (Table 3). In contrast, no virulence genes were detected in P1 water at all sampling times as well as in the water of the remaining ponds (P2, P3, and P4) at non-disease sampling times.

### Bacterial co-occurrence networks

**Figure 4:**
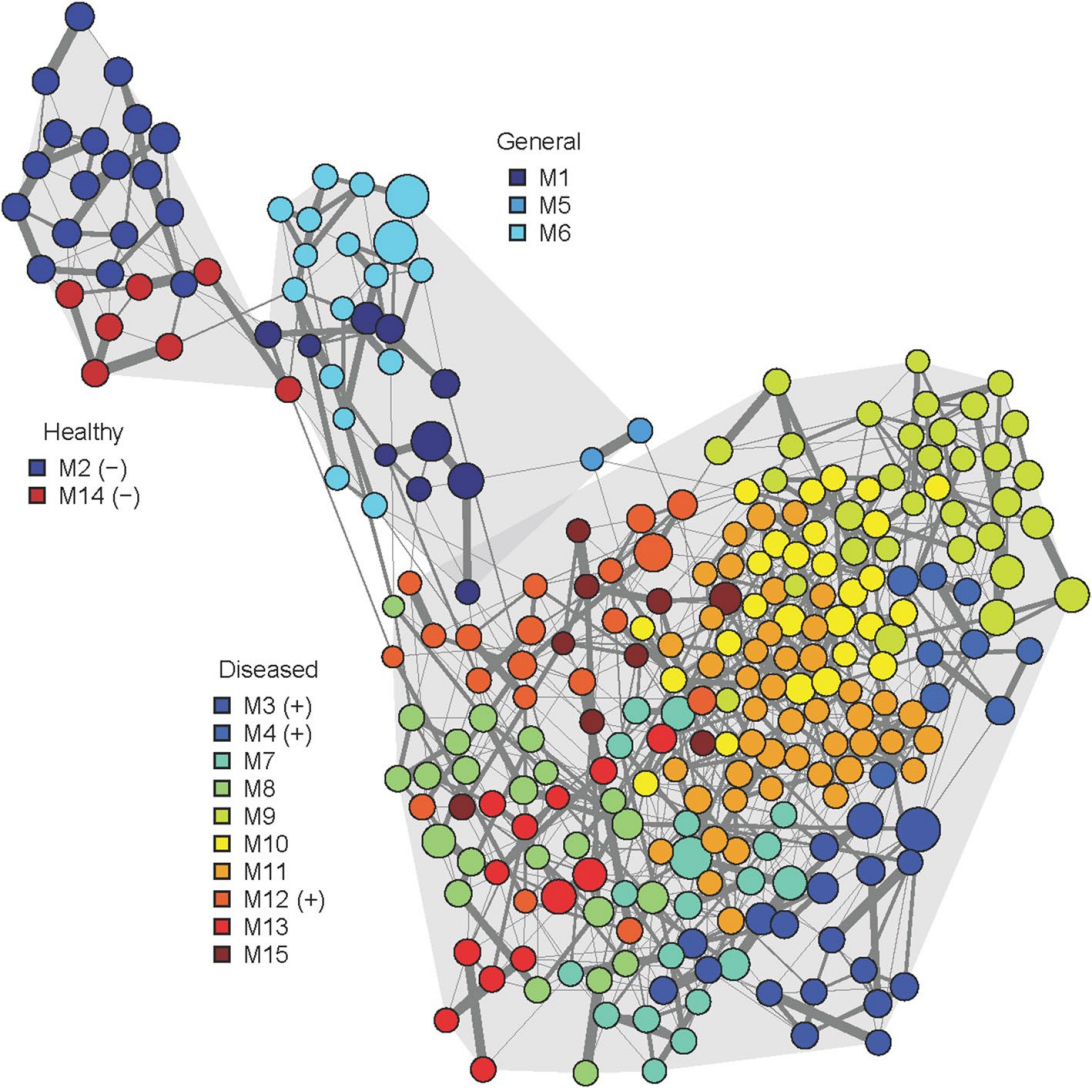
Bacterial co-occurrence network generated by SPIEC-EASI. Node size corresponds to the average sequence proportion of operational taxonomic units in intestinal and faecal samples. Network modules detected by Louvain clustering are shown in different colours, grouped by the samples they predominantly occurred in: healthy, diseased, both (general). Network modules identified as characteristic for healthy and diseased shrimps by random forest models are indicated by (−) and (+), respectively. Edge width corresponds to the strength of the association between OTUs.

**Figure 5:**
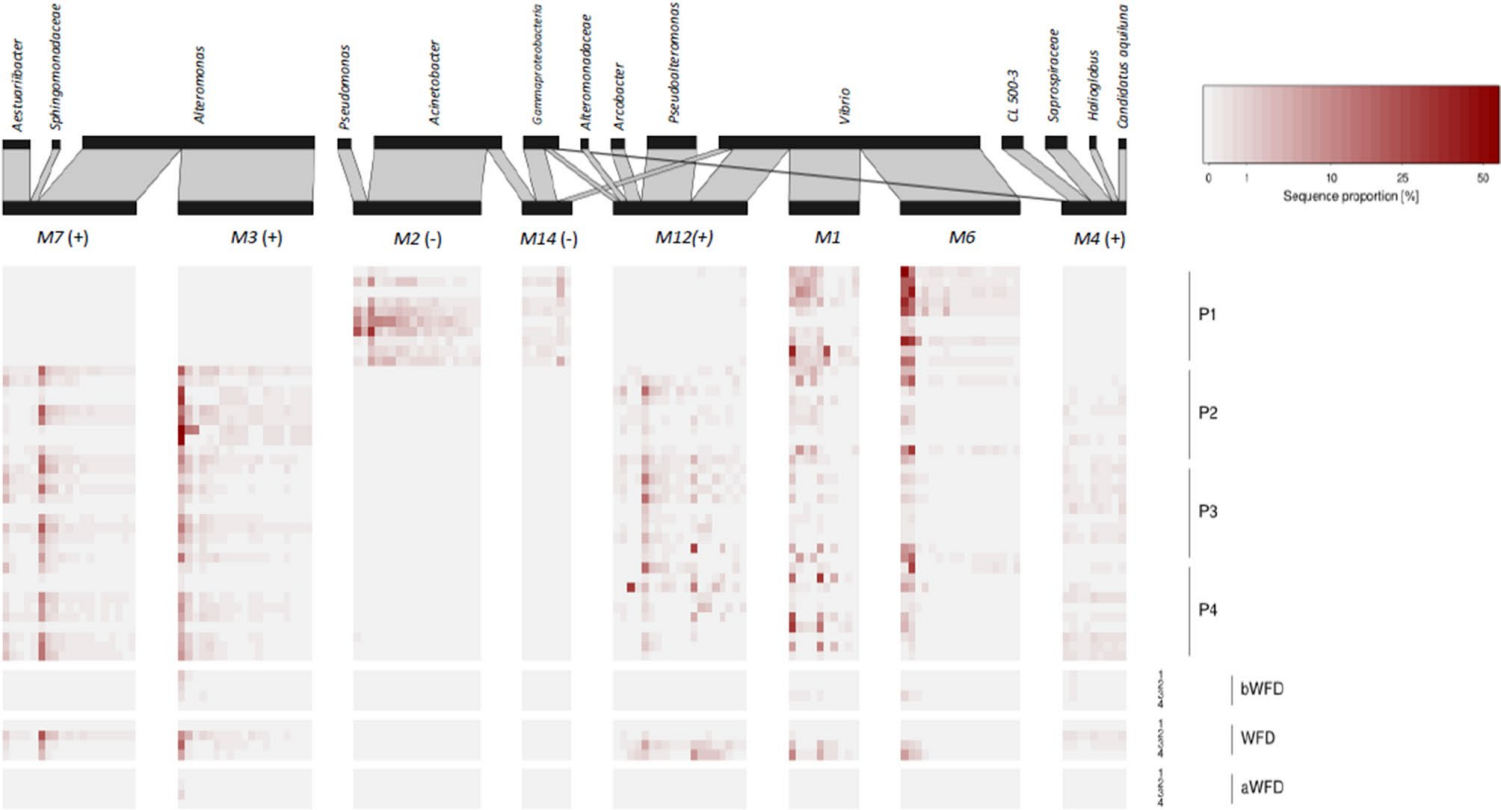
Sequence proportion of the members of the most dominant and most distinguishing network modules between healthy (−) and diseased shrimps (+), as well as their contribution to the particle-associated fraction from the respective ponds and sampling times. Their taxonomic affiliation is provided on genus level. Water samples were not used for the network construction.

After filtering rare and low sample coverage OTUs, 269 OTUs were retained from IB and FSB samples for co-occurrence network analysis using sparse inverse covariance estimation for ecological association inference (SPIEC-EASI). Louvain clustering was able to generate 15 bacterial co-occurrence modules (Fig. 4 and Supplementary Table 4). Network modules with highest sequence proportions of their member OTUs in shrimp and PA bacterial community samples were visualized in a heatmap (Fig. 5). Among 15 modules, two modules (M2 and M14) represented co-occurring OTUs unique to IB samples of healthy shrimps. They consisted of *Acinetobacter*, *Pseudomonas*, as well as two *Vibrio* OTUs. Interestingly, these *Acinetobacter*, *Pseudomonas*, and *Vibrio* OTUs were absent in all WB including those from P1. OTUs represented in modules 1, 5, and 6 occurred in both healthy and diseased shrimps, and were exclusively affiliated with *Vibrio* (M1, M6) and *Photobacterium* (M5). Ten modules (M3, M4, M7, M8, M9, M10, M11, M12 and M15) comprised of OTUs predominantly found in infected shrimps. Module 3 consisted exclusively of *Alteromonas* OTUs, while the remaining modules consisted of more than three genera. Notably, *Vibrio* OTUs appeared in both healthy and diseased shrimp samples, and contributed to general network modules (M1, M6), as well as those characteristic for either healthy (M14) or diseased shrimps (M12), although associated with different other taxa. For instance, in M12 *Vibrio* co-occurred with *Arcobacter* and *Pseudoalteromonas*, while in M14 *Vibrio* OTUs were associated with *Acinetobacter*. Pairwise random forest models were further used to select network module best suited to distinguish diseased from healthy shrimp samples based on mean decrease Gini and accuracy (Supplementary Table 5). Random forests confirmed M2 and M14 as most characteristic for healthy, and M3, M4, M12 for diseased shrimp samples.

## Discussion

To better understand WFD in *Penaeus vannamei* aquaculture, we measured water quality and analysed bacterial community dynamics. Based on the visual estimation of white faecal string (FS) numbers in the ponds, we discriminated the WFD event into two phases: start of disease (early symptoms), represented by P3 and P4, with lower numbers of white FS, and early-outbreak (P2), with greater white FS numbers. Because bacterial communities of fresh shrimp faeces and that of the full intestines of healthy *P. vannamei* have been shown to be comparable^17,34^, we only dissected the intestines of healthy shrimps and analysed them together with the fresh faecal strings collected from diseased shrimp. In addition, if the shrimp already defecated, it was difficult to distinguish healthy and infected shrimps since the shrimp intestine was already empty.

Water quality has a large impact on the health status and growth of the shrimps^21^ as well as on the BCC in shrimp pond waters^36^. Regular feed input causes unintended negative effects on water quality, which eventually limit shrimp growth. Uneaten feed pellets, which are not incorporated by shrimps, together with organic matter waste (i.e. faeces) stimulate phytoplankton and bacterial growth resulting in bacterioplankton community instability^37^. Elevated metabolic activity due to a heterotrophic bacterioplankton bloom exerts an increased oxygen demand, and influences other physical parameters such as the amount of suspended particulate matter and turbidity^38^ as well as inorganic nutrient concentrations^39^. Microbial activities including organic matter degradation, respiration and nitrification process, and accumulation of dissolved carbon dioxide will affect hydrogen ion concentration in pond water resulting in decrease of pH and alkalinity^38^, as was observed in ponds with diseased shrimps. In contrast, external intervention by regular addition of lime stone which may rich of calcium carbonate and reactive silicate may buffer pH and alkalinity level^38^, which was the case in the pond with healthy shrimps.

A salinity range of 32.7–34.6 psu in shrimp pond water favoured the dominance of marine heterotrophic bacteria. At non-disease events, *Exiguobacterium*, *Halomonas*, *Psychrobacter*, *Salegentibacter* and *Sulfitobacter* dominated the bacterial communities in pond water (WB), presumably playing a role in nitrification^40–42^, organic matter degradation and sulphite oxidation^43^. They may also inhibit the growth of potential pathogenic bacteria in pond water, for example *Pseudoalteromonas* and *Vibrio*, as reported in previous studies^16,35,44^. Furthermore, intestinal bacteria (IB) of the healthy shrimps were dominated by *Acinetobacter*, *Pseudomonas* and *Vibrio* which correspond to those reported by previous studies^15,33,34^. Interestingly, *toxR* and *tlh* genes belonging to *V. parahaemolyticus* were detected in similar concentrations in the intestines of healthy and diseased shrimps. Thus, we predict that *Acinetobacter*, *Pseudomonas* and other *Vibrio* may inhibit the pathogenicity of *V. parahaemolyticus*. These seemingly beneficial bacterial taxa are known to drive nitrification processes, accumulate poly-ß-hydroxybutirate (PHB) which may stimulate the growth of beneficial bacteria, and act as antagonistic bacteria against pathogens^13,26,32,45–48^. For instance, they can inactivate acyl-homoserine lactone (AHL), a type of quorum sensing molecule, which regulates the virulence of pathogenic bacteria^48^. Furthermore, the IB differed considerably from WB at non-disease events. Since *Acinetobacter* and *Pseudomonas* are intolerant to high salinity^46,47^, we propose that they cannot persist in the saline shrimp pond water. Therefore, they did not enrich WB, resulting in the observed high community dissimilarities.

Our study indicates that a pulse disturbance^49^, such as a sudden decrease of pH (below 8) and dissolved oxygen (below 6 mg L^−1^), and an increase of inorganic nutrients as observed in P2-P4, may affect shrimps and bacterial communities in shrimp pond waters (WB). The pulse disturbance caused stress in shrimps, which may in turn have induced changes in the intestinal bacterial communities, resulting in opportunistic pathogenic bacteria, such as *Alteromonas*, *Marinomonas*, *Photobacterium*, *Pseudoalteromonas* and *Vibrio*, becoming dominant in the bacterial communities in white faecal strings (FSB). At this stage, we deduce that dysbiosis in the IB, which was also reported in previous WFD related studies^22,50^, had occurred. We observed a gradual shift from presumably beneficial bacteria-dominated to potential pathogen-dominated FSB, which coincided with the progression of the disease from the ponds with early symptoms to the pond at early outbreak. This suggests that changes in intestinal bacterial communities may be closely associated with the severity of the shrimp disease. This hypothesis is supported by a previous studies^17^, which reported that changes in shrimp intestinal bacteria occurred in parallel with changes in disease severity, reflecting the transition from a healthy to a diseased state. Among the potential pathogenic taxa, which dominated FSB communities in our study, *Photobacterium*, *Pseudoalteromonas* and *Vibrio* corresponded to those previously observed to be associated with the WFD events^22^. However, some genera such as *Aeromonas, Candidatus* Bacilloplasma, *Phascolarctobacterium* and *Staphylococcus*, which were reported to be present in previous study^20,22^, were absent in our samples during the WFD event. It is important to consider, though, that geographical location, shrimp farm management, and different methodological approaches may influence the detection of bacterial taxa.

Shifts of WB occurred in both FL and PA fractions during the disease events, which coincided with decreased pH*.* We propose that lower pH altered growth rates of heterotrophic bacteria, as also reported previously^51^ resulting in a dominance of opportunistic, potentially pathogenic bacteria such as *Alteromonas*, *Pseudoalteromonas* and *Vibrio* in WB. Since shrimp faeces easily disintegrate in the pond water (up to 27% within 12 h)^34^, and could be unravelled faster due to water movement and mechanical aeration, we suggest that FSB enriched WB, thereby contributing to the dominance of *Alteromonas* in FL and PA, as observed in the WB of P2. Disintegration of faeces will facilitate bacterial dispersion, as well as protein and inorganic nutrient enrichment from faeces^34^. The enrichment of the WB by opportunistic pathogenic bacteria further seemed to correlate with disease severity and the number of infected shrimps. This is reflected in the significantly higher concentrations of *toxR* and *tlh* genes in pond water samples from the early outbreak phase compared to the ponds with early symptoms. Furthermore, if greater numbers of pathogenic bacteria are released in the pond water and incorporated into particulate matter, it will accelerate the spread of the disease among shrimps, since healthy shrimps may consume pathogen-laden particles and become intoxicated. Thus, in this scenario, FSB not only contribute to bacterial abundance, structure and function of the WB, but also enforce a detrimental feed-back on shrimp health.

The infection of shrimp tissue is caused by the production of haemolysins by pathogenic bacteria (e.g. *V. parahaemolyticus*) upon activation of their virulence factor genes^52–54^. However, their ability to provoke disease is dependent on abiotic (e.g. pH, salinity and temperature) and biotic (e.g. bacterial co-occurrence) factors that support their outbreak^55,56^. We explored such biotic interactions using bacterial co-occurrence networks. Assemblages of co-occurring OTUs of healthy shrimps could be clearly distinguished from those of diseased shrimps. We propose that *Acinetobacter* and *Pseudomonas* composing network module 2, as well as *Acinetobacter* and the two *Vibrio* OTUs composing network module 14 are part of the indigenous beneficial bacterial community of the healthy shrimps. The detection of *Vibrio* OTUs in both healthy and infected shrimps and in inversely correlated co-occurrence modules suggests the presence of different *Vibrio* strains with contrasting interactions. While some *Vibrio* OTUs might represent opportunistic pathogens, others may even be beneficial in low proportions^57,58^. Alternatively, the co-occurrence with other bacteria such as *Acinetobacter* may prevent the activation of virulence factor genes, despite the presence of potentially pathogenic *Vibrio* in the intestines of healthy shrimps. Conversely, the change in *Vibrio*-associated co-occurrence patterns in diseased shrimps from presumably beneficial to other opportunistic and potentially also pathogenic taxa (network module 12), may contribute to the disease outbreak.

Considering differences of IB communities of healthy shrimps and WB at non-disease event from those of WFD samples, as well as co-occurrence patterns in healthy and diseased shrimp samples, we highlight that the dysbiosis in IB and a shift from halophilic bacteria-dominated to pathogenic bacteria-dominated in pond waters contribute to the aetiology of the studied WFD outbreak. We emphasize that immediate re-adjustment of water quality parameters, specifically adjusting pH to above 8, will allow WB to return to its pre-disturbance composition and terminate the outbreak, followed by recovery from WFD, as indicated by the lack of symptoms and detectable virulence genes in WB, and no shrimp mortality. This implies a resilience of bacterial communities in shrimp pond water after short disturbances, as can also be observed in other environments^49,59,60^. However, we point out that prolonged exposure to water deterioration and elevated pathogen proportions may increase disease severity and lead to mass mortality of cultured shrimps as previously observed^5,61^. Our findings on the application of commercial probiotics to cure WFD in shrimps revealed that probiotic bacteria such as *Lactobacillus* were absent in WB, IB and FSB, suggesting that such an application was not effective. *Lactobacillus* was no longer detectable after they were diluted in the shrimp pond water. Instead of spreading the probiotics into the pond water, we propose to add them to the feed pellets, which will be eaten by shrimps. With this method, colonization of probiotic bacteria in the shrimp intestine may occur more effectively.

In conclusion, environmental stressors, specifically a decrease in pH and dissolved oxygen, induced a substantial community shift in WB and affected shrimp physiology, which in turn resulted in changes of the intestinal bacterial community and subsequently the emergence of WFD. Moreover, we report several opportunistic bacterial taxa such as *Arcobacter*, *Alteromonas, Marinomonas, Photobacterium* and *Pseudoalteromonas*, which may contribute to or even cause WFD. To avoid shrimp loss, shrimp farming management should focus on maintaining sediment/sludge and water quality (i.e. pH, dissolved oxygen, turbidity, inorganic nutrients and SPM) as well as promoting a stable intestinal bacterial community composition, where beneficial bacteria, even in low proportions, are able to inhibit the pathogenicity of *Vibrio*.

## Materials and methods

### Sample collection and sampling sites

Water samples were collected between 9 and 11 a.m. from one pond with healthy shrimps (P1, which served as control) and 3 shrimp ponds (P2, P3, and P4) that experienced a WFD event between 50 to 70 days of rearing in October–November 2016. All ponds were lined with high density polyethylene (HDPE) plastic and chlorinated 2 weeks before shrimp rearing. Initial population densities were 40 (P2) and 90 post-larvae m^−3^ (P1, P3, and P4), with the same origin of shrimp fries (PL15, specific pathogen free, Central Pertiwi Bahari Firm, Rembang, Central Java, Indonesia). Shrimp ponds were located in Rembang Regency, Central Java, Indonesia (6°37′41.13″ S 111°30′1″ E and 6°42′11.66″ S 111°21′54″ E). Water sampling as well as measurements of environmental parameters were described in a previous study^35^. Environmental data were deposited on PANGAEA (https://doi.pangaea.de/10.1594/PANGAEA.908247).

For bacterial community analysis, ten fresh white faecal strings were collected from feeding trays of each pond with infected shrimps. Ten healthy shrimps from P1 were collected using the feeding tray and put on ice in the cold storage immediately. They were then dissected in the laboratory to retrieve their filled intestines with sterile dissecting tools. Before dissection, shrimps were swabbed with ethanol 70% to sterilize their body and to avoid contamination from the carapace. All samples were immediately put in Eppendorf tubes, frozen and stored at − 20 °C until DNA extraction.

### Culturable presumptive pathogenic bacterial strain enumeration and identification from pond water

To obtain culturable presumptive pathogenic bacteria (*Vibrio*) from all ponds, 100 µL of undiluted to 10^–4^ diluted water sample were plated onto selective thiosulfate citrate bile salts sucrose (TCBS) medium (Roth, Karlsruhe, Germany), followed by incubation at 35 °C for 24 h. Colonies which grew on the TCBS media were then counted to determine culturable presumptive pathogenic *Vibrio* numbers. Strains which grew on TCBS plates from P1 at 60th day sampling were pooled by swabbing and collected into Eppendorf tubes containing 100 µl sterile sea water, and stored at − 20 °C until DNA extraction and sequencing-based taxonomic analysis. In total, colonies from 6 TCBS plates were pooled into 1 Eppendorf tube per plate.

### Molecular analysis of bacterial communities

500 mL of water samples were filtered to collect bacterial cells. To distinguish between free-living (FL) and particle-associated (PA) bacterial communities, a serial filtration was conducted through 3.0 µm and 0.2 µm polycarbonate filters (ø 47 mm, Whatman, Dassel, Germany) for the PA and the FL bacterial fractions, respectively. Genomic DNA from water samples was extracted according to Nercessian et al.^62^, while bacterial cells from intestines, white faecal strings, and isolates were extracted using phenol–chloroform methods^63^. DNA pellets were dissolved in 40 µl TE buffer (10 mM Tris–HCl, 1 mM EDTA, pH 8.5). DNA concentrations were measured photometrically and checked for purity (ratio of light absorption at 260 to 280 nm) using a nanoquant plate reader (Infinite M200 Pro, Tecan, Germany). Filtration, DNA extraction as well as genomic DNA concentration measurements were done in triplicates.

16S rRNA gene amplification was performed from genomic DNA extracts. DNA sequences of the V3-V4 hypervariable region of the 16S rRNA gene were obtained from amplicon sequencing with the primer set S-D-Bact-0314-b-S-17 (5′-CCTACGGGNGGCWGCAG-3′)/S-D-Bact-0785-a-A-21 (5′-GACTACHVGGGTATCTAAKCC-3′)^64^. Sequencing at LGC genomics (Berlin, Germany) was performed on an Illumina MiSeq using the V3 Chemistry (Illumina) in a 2 × 300 bp paired-end run. Demultiplexing, i.e. grouping of sequences by sample, and the removal of the primer sequences from the raw paired-end reads were performed by LGC genomics (Berlin, Germany). Sequences from genomic DNA from water samples before and after the disease period in P2, P3, and P4, as well as P1 at rearing days 50, 60, and 70 were retrieved from a previous study (PRJEB26390)^35^.

Sequences were quality-trimmed with a sliding window of four bases and a minimum average quality of 15 with *trimmomatic* v.033^65^. Quality trimmed sequences were merged using PEAR v0.9.8^66^. Then, Minimum Entropy Decomposition (MED) was used to cluster sequences into OTUs^67,68^. MED applies the principle of oligotyping^67^, which uses the Shannon entropy to iteratively partition amplicons at single nucleotide resolution, thereby providing more accurate descriptions of closely related but distinct taxa^69^. During MED, we used an entropy threshold of 0.0965 and a minimum substantive abundance (-M) of 50 to avoid the generation of low abundant OTUs, decomposing the data set one nucleotide position at a time (-d 1). For each OTU (oligotyping node), one representative sequence was taxonomically classified with SINA (SILVA Incremental Aligner) v1.2.11 using the SILVA rRNA project reference database (SILVA version 132) at a minimum alignment similarity and quality of 0.9 and a last common ancestor consensus of 0.7^70^. Unwanted lineages (such as archaea, chloroplasts, and mitochondria) were removed. In order to obtain results comparable to the previously generated data^35^ for WB analysis, OTU profiles from independently sequenced triplicate samples of the FL and PA fractions of P2, P3, and P4 at the WFD event were merged by taking the sum of the sequence counts per OTU.

### Detection and quantification of virulence genes

Three virulence factor genes belonging to *Vibrio* which are transcriptional regulator (*toxR*)*,* thermolabile haemolysin (*tlh*), and thermostable direct haemolysin (*tdh*) were checked in a quantitative PCR machine (CFX Connect Real-time System Bio-Rad, München, Germany) using the primer sets described previously^35^. qPCR conditions were as follows: a reaction mixture consisted of 10 µL 2X SensiFast SYBR No-ROX (Bioline, Luckenwalde, Germany), 1 µL of 25 mM MgCl_2_ (Roboklon EURx, Berlin, Germany), 0.2 µL of 0.5 mM forward and reverse primer (Biomers, Ulm, Germany), 8.8 µL sterile distilled water, and 2 µl of DNA template (concentration 0.5–10 ng µL^−1^). The 3-step qPCR amplification was performed as follows: pre-denaturation at 95 °C for 3 min, followed by 40 elongation cycles consisting of denaturation at 95 °C for 10 s, annealing at 60 °C for 15 s, and extension at 72 °C for 20 s, and a dissociation step after final elongation was added to improve amplification specificity. *V. parahaemolyticus* DSM 11058 (DSMZ, Braunschweig, Germany) was used as positive control for *toxR, tlh,* and *tdh* genes, while *V. vulnificus* DSM 10143 (DSMZ, Braunschweig, Germany) served as negative control. A serial dilution of the positive control (known concentration) was used to estimate gen copy numbers from environmental samples (Supplementary Information Table 3). Gene copy numbers for *toxR* and *tlh* were determined with the equation y = − 3.554x + 44.891 with R^2^: 0.994 and y = − 3.300x + 42.982 with R^2^: 0.996, respectively.

### Data analysis

A principal component analysis (PCA) was conducted to examine the relationship among environmental parameters and to characterize shrimp ponds during the WFD outbreaks. DNA sequence samples were categorized into WB (12 PA and 11 FL samples), shrimp bacteria, i.e. IB and FSB (10 and 30 samples, respectively), culturable *Vibrio* strains from the pond with healthy shrimp (6 samples), and probiotic bacteria (1 sample). BCC patterns in all samples were visualized by non-metric multidimensional scaling (NMDS) based on Bray–Curtis dissimilarities, while pairwise ANOSIM tests applying Benjamini–Hochberg p-value correction were performed to detect separation of bacterial communities between ponds for IB and FSB samples. Changes in Bray–Curtis dissimilarities between FSB to WB of each diseased pond before, during, and after the disease event were compared using Kruskal–Wallis rank sum tests, followed by pairwise Wilcoxon tests with Benjamini–Hochberg p-value correction. Kruskal–Wallis rank sum tests was performed because Bray–Curtis dissimilarity values were not normally distributed.

Differences in the concentrations of *toxR* and *tlh* genes among ponds were tested using ANOVA for shrimp, and MANOVA for water samples to account for the dependence of observations from FL and PA fractions. Individual ANOVAs were performed per fraction once MANOVA indicated a difference in gene copy numbers between the FL and the PA fractions, followed by multiple pairwise comparisons (TukeyHSD post-hoc tests) to assess difference between ponds.

OTUs from intestine and white faecal string (FS) were analysed to identify sub-populations (modules) of co-occurring bacteria using SPIEC-EASI (Sparse inverse covariance estimation for ecological association inference) version 1.0.2^71^. The statistical method SPIEC-EASI comprises two steps, first a transformation for compositionality correction of the OTU matrix, and second an estimation of the interaction graph from the transformed data using sparse inverse covariance selection^71^. Pre-filtering of OTUs was performed before SPIEC-EASI to exclude rare and low sample-coverage OTUs, retaining only OTUs which occurred in at least five samples with a proportion of least 0.1%. Regression coefficients from the SPIEC-EASI output were extracted and used as edge weights to generate a bacterial co-occurrence network using *igraph*^72^. Negative edge weights, which indicated inverse trends among OTUs were excluded for Louvain clustering, which was then performed to extract network modules. Modules characteristic for the IB of the healthy pond and the FSB of each of the diseased ponds were identified using pairwise random forest models based on module eigengenes. Module eigengenes and random forests models were calculated using the R packages WGCNA^73^ and randomForest^74^, respectively. The sequence proportions of the members of the modules related to healthy shrimp or the WFD events (based on the highest mean decrease Gini and accuracy) were visualized in a heatmap.

All statistical analyses, as well as figure visualizations were performed in R (R version 3.4.2, R Core Team, 2017, using R Studio v.0.98.1056) with the packages vegan^75^, nlme^76^, gplots^77^ and packages mentioned previously.

## Supplementary information

Supplementary file1 (PDF 298 kb)

## Data Availability

DNA sequences generated in this study were deposited on ENA with accession number PRJEB37200 (https://www.ebi.ac.uk/ena/data/view/PRJEB37200), while biogeochemical parameters and R scripts for statistical analyses were submitted to PANGEA (https://doi.pangaea.de/10.1594/PANGAEA.908247) using the data brokerage service of the German Federation for Biological Data/GFBio^78^ in compliance with the Minimal Information about any (X) Sequence (MIxS) standard^79^.
