# Supplementary material for: Structure and co-occurrence patterns of bacterial communities associated with white faeces disease outbreaks in Pacific white-leg shrimp Penaeus vannamei aquaculture
Source: Sci Rep. 2020 Jul 20;10:11980. doi: 10.1038/s41598-020-68891-6 (PMC7371890; doi:10.1038/s41598-020-68891-6)
Supplement: Supplementary file 1 — Supplementary file1 (PDF 298 kb) [file 41598_2020_68891_MOESM1_ESM.pdf]

Supplementary Information

Structure and co-occurrence patterns of bacterial communities associated with white faeces disease outbreaks in Pacific white-leg shrimp *Penaeus vannamei* aquaculture

Yustian Rovi Alfiansah[1,2,3*], Sonja Peters[1], Jens Harder[4], Christiane Hassenrück[1] and Astrid Gärdes[1,5,6]

[1] Leibniz Centre for Tropical Marine Research (ZMT), Bremen, 28359, Germany
[2] Research Center for Oceanography (RCO-LIPI), Jakarta, 14430, Indonesia
[3] Center for Aquaculture Research (ZAF), Alfred Wegener Institute (AWI), Bremerhaven, 27570 Germany
[4] Department of Molecular Ecology, Max Planck Institute for Marine Microbiology (MPI-MM), Bremen, 28359 Germany
[5] Division Biosciences/Polar Biological Oceanography, Alfred Wegener Institute (AWI), Bremerhaven, 27570 Germany

[6] Hochschule (HS) Bremerhaven, Bremerhaven, 27568 Germany

Correspondence and requests for materials should be addressed to YRA

(Corresponding author: yustian.alfiansah@leibniz-zmt.de)

Supplementary Information Table 1. Bray-Curtis dissimilarity based on relative OTU proportions between WB communities before, during and after the WFD event and the FSB of the respective ponds during the WFD event.

Ponds	Periods	Min-Max		Average		Kruskal Wallis	
		FL	PA	FL	PA	FL	PA
2	before WFD	0.89-0.93	0.90-0.99	0.92[a]	0.97[a]	$\chi = 21.93$	$\chi = 20.60$
	during WFD	0.55-0.88	0.42-0.86	0.75[b]	0.70[b]	df= 2	df= 2
	after WFD	0.87-0.95	0.95-0.98	0.93[a]	0.97[a]	$p = < 0.01$	$p = < 0.01$
3	before WFD	0.85-0.96	0.95-0.98	0.92[a]	0.96[a]	$\chi = 14.22$	$\chi = 20.20$
	during WFD	NA	0.55-0.91	NA	0.67[b]	df= 1	df= 2
	after WFD	0.84-0.98	0.76-0.99	0.90[a]	0.95[a]	$p = 0.762$	$p = < 0.01$
4	before WFD	0.93-0.99	0.96-1	0.95[a]	0.98[a]	$\chi = 19.89$	$\chi = 19.35$
	during WFD	0.66-0.93	0.53-0.84	0.80[b]	0.74[b]	df= 2	df= 2
	after WFD	0.96-0.99	0.96-1	0.98[a]	0.98[a]	$p = < 0.01$	$p = < 0.01$

WFD: white faeces disease event, NA: not applied, FL: free-living, PA: particle-associated fractions. Superscript letter indicate significant differences based on pairwise Wilcoxon Wilcoxon tests with Benjamini-Hochberg p-value correction.

Supplementary Information Table 2. Genomic DNA concentrations and estimations of virulence gene concentrations (copy numbers) in *V. parahaemolyticus* from two cell collection assays.

Bacterial Culture	Cell numbers (cell mL^{-1})	DNA concentration* (ngμL^{-1})	*toxR*		*tlh*		*tdh*	
			Copy numbers per ml culture **	Cycle (ct)	Copy numbers per ml culture **	Cycle (ct)	Copy numbers per ml culture **	Cycle (ct)
V. parahaemolyticus DSM 11058	2.28 x 10^{8}	[a]Filtration:						
		I. 155.1	4.45 x 10^{8}	14.59	4.45 x 10^{8}	14.80	4.45 x 10^{8}	14.55
		II. 93.9	2.69 x 10^{8}	14.89	2.69 x 10^{8}	15.12	2.69 x 10^{8}	15.37
		III. 62.0	1.78 x 10^{8}	14.61	1.78 x 10^{8}	14.88	1.78 x 10^{8}	14.95
		[b]Cells:						
		I. 690.2	6.20 x 10^{9}	15.24	NA	NA	NA	NA
		II. 154.7	1.34 x 10^{9}	14.65	NA	NA	NA	NA
		III. 616.6	5.55 x 10^{9}	14.82	NA	NA	NA	NA

*DNA yield of extracted *V. parahaemolyticus* culture from two cell collection/preparation assays. 1st assay ([a]): 5mL cell suspension were filtered on a polycarbonate filter (pore size: 0.2 µm, diameter: 47 mm), and the DNA extract was eluted in 80 µl of TE buffer. 2nd assay ([b]): 2 mL cell suspension was centrifuged, and DNA extract was eluted in 100 µL of TE buffer.

Copy number calculation according to an equation from ThermoFischer scientific (https://www.thermofisher.com/de/de/home/brands/thermo-scientific/molecular-biology/molecular-biology-learning-center/molecular-biology-resource-library/thermo-scientific-web-tools/dna-copy-number-calculator.html, date accessed 15.09.2019) where molar mass is 650 $gmol^{-1}$ per base pair (bp), fragment length (nt) is 5165770 (total genome of *V. parahaemolyticus*) resulting in a concentration of 1.68 x 10^{7} gene copy per ng μL^{-1}DNA extract. Measured concentrations (copy numbers) were calculated by multiplication of total volume of the DNA extract and copy number per ng μL^{-1}DNA extract and then divided by volume of the bacterial culture. Underlined value in DNA concentration indicated the genomic DNA which was used for qPCR test and gene copy quantification. All virulence genes are located in chromosome II having single copy per *V. parahaemolyticus* cell (Makino *et al*. Genome sequence of *Vibrio parahaemolyticus*: a pathogenic mechanism distinct from that of *V. cholera*, The Lancet **361, 743-749 (2003)).

Supplementary Information Table 3. Gene copy numbers from serial dilution of the *V. parahaemolyticus* (*Vp)* DNA as positive control for the detection of *toxR*, *tlh*, and *tdh* gene.

Dilution	DNA concentration (ngµL^{-1})	Estimation of *Vp* cell	*toxR*		*Tlh*		*tdh*	
			Copy numbers (log copy number mL^{-1})	Cycle (ct)	Copy numbers (log copy number mL^{-1})	Cycle(ct)	Copy numbers (log copy number mL^{-1})	Cycle(ct)
10^0	93.9	2.28 x 10^8	8.43	14.9	8.43	14.9	8.43	14.9
10^{-1}	9.39	2.28 x 10^7	7.43	17.7	7.43	17.8	7.43	17.0
10^{-2}	9.39 x 10^{-1}	2.28 x 10^6	6.43	21.7	6.43	22.0	6.43	20.9
10^{-3}	9.39 x 10^{-2}	2.28 x 10^5	5.43	25.2	5.43	25.8	5.43	24.9
10^{-4}	9.39 x 10^{-3}	2.28 x 10^4	4.43	28.6	4.43	28.8	4.43	29.4
10^{-5}	9.39 x 10^{-4}	2.28 x 10^3	3.43	31.9	3.43	31.9	3.43	33.3
10^{-6}	9.39 x 10^{-5}	2.28 x 10^2	2.43	34.8	2.43	34.9	2.43	36.7
10^{-7}	9.39 x 10^{-6}	2.28 x 10^1	1.43	37.9	1.43	37.8	< LoQ	< LoD

LoQ: Limit of quantification, LoD: Limit of detection

Supplementary Information Table 4. Modules generated by Louvain clustering.

Modules	Members (OTUs)
1	*Vibrio* (10)
2	*Acinetobacter* (16), *Pseudomonas* (2)
3	*Alteromonas* (19)
4	*Candidatus aquilina* (1), CL 500-3 (3), *Gammaproteobacteria*_unclassified (1), *Halioglobus* (1), *Saprospiraceae*_unclassified (3)
5	*Photobacterium* (2)
6	*Vibrio* (17)
7	*Aestuariibacter* (4), *Alteromonas* (14), *Sphingomonadaceae*_unclassified (1)
8	*Alteromonadaceae*_unclassified (5), *Gammaproteobacteria*_unclassified (2), *Pseudoalteromonas* (6), *Psychrosphaera* (3), *Salinomonas* (1), *Vibrio* (4), *Vibrionaceae*_unclassified (2)
9	*Alteromonas* (1), *Caldilineaceae*_unclassified (1), *Cyanobium* PCC-6307 (1), GWA2-38-13b_unclassified (2), *Halomonas* (3), *Ilumatobacteraceae*_unclassified (2), *Marivita* (2), *Microbacteriaceae*_unclassified (3), *Nautella* (1), *Nitriliruptoraceae*_unclassified (8), PeM15_unclassified, *Pirellulaceae*_unclassified (1), *Planctomicrobium* (1), *Rhodobacteraceae*_unclassified (2), *Ruegeria* (1), *Synechococcus* cc9902 (1)
10	*Aestuariibacter* (1), *Alphaproteobacteria*_unclassified (1), *Candidatus actinomarina* (1), CHAB-XI-27_unclassified (1), Clade III_unclassified (1), Crocinitomix (1), Flavobacteriaceae_unclassified (3), Halioglobus (6), HIMB11 (1), *Idiomarina* (1), *Marivita* (1), *Nitriliruptoraceae*_unclassified (1), OM43 clade (1), *Peredibacter* (1), *Rhizobiaceae*_unclassified (1), *Rhodobacteraceae*_unclassified (1), *Robiginitalea* (1), *Salinihabitans* (1), Sva0996 marine group (1), *Winogradskyella* (1)
11	*Alteromonas* (1), *Aurantivirga* (1), *Bacteroidia*_unclassified (1), *Balneola* (1), *Balneolaceae*_unclassified (1), *Candidatus actinomarina* (1), *Chitinophagales*_unclassified (2), *Crocinitomicaceae*_unclassified (1), *Cryomorphaceae*_unclassified (3), *Flavobacteriaceae*_unclassified (7), *Kiloniella* (1), Litoricola (2), *Marinagarivorans* (1), NS11-12 marine group_unclassified (2), NS7 marine group_unclassified (1), NS9 marine group_unclassified (1), *Oleibacter* (3), *Oleiphilus* (1), *Owenweeksia* (3), *Phaedactylibacter* (1), *Pseudoalteromonas* (1), *Rhodothermaceae*_unclassified (1), *Saccharospirillaceae*_unclassified (3), *Saprospiraceae*_unclassified (2), SAR 324 clade (Marine group B)_unclassified (1), SM2D12_unclassified (1), *Tenacibaculum* (1)
12	*Alteromonadaceae*_unclassified (1), *Arcobacter* (2), *Gammaproteobacteria*_unclassified (1), *Pseudoalteromonas* (7), *Vibrio* (8)
13	*Alteromonadaceae*_unclassified (1), *Marinomonas* (7), *Pseudoalteromonas* (1), *Salinomonas* (3), *Vibrio* (1)
14	*Acinetobacter* (2), *Gammaproteobacteria*_unclassified (1), *Vibrio* (2)
15	*Aestuariibacter* (1), *Alteromonadaceae*_unclassified (1), *Gammaproteobacteria*_unclassified (1), *Pseudoalteromonas* (5), *Vibrio* (1)

Supplementary Information Table 5. Importance values of each module in pairwise Random Forest models between P1 (pond with healthy shrimps) and each of the ponds with diseased shrimps.

Modules	P1-P2		P1-P3		P1-P4	
	MDA	MDG	MDA	MDG	MDA	MDG
1	0.010	0.441	0.012	0.523	0.003	0.319
**2**	**0.078**	**1.586**	**0.072**	**1.490**	**0.086**	**1.720**
**3**	**0.076**	**1.527**	0.016	0.461	0.008	0.366
**4**	**0.029**	**0.815**	**0.026**	**0.706**	**0.078**	**1.637**
5	<0.001	0.032	0.0003	0.055	<0.001	0.044
**6**	**0.018**	**0.669**	**0.017**	**0.615**	**0.020**	**0.736**
**7**	**0.006**	**0.263**	**0.033**	**0.823**	**0.008**	**0.336**
8	0.015	0.544	0.002	0.077	0.006	0.185
9	0.030	0.843	0.029	0.798	0.023	0.898
10	0.006	0.352	0.027	0.865	0.018	0.531
11	0.005	0.186	0.004	0.137	0.013	0.462
**12**	**0.014**	**0.411**	**0.040**	**1.098**	**0.010**	**0.357**
13	0.005	0.142	0.002	0.087	0.003	0.114
**14**	**0.080**	**1.550**	**0.080**	**1.600**	**0.078**	**1.611**
15	0.002	0.137	0.005	0.165	0.003	0.183

MDA: Mean decrease accuracy, MDG: Mean decrease gini, Module highlighted in bold are shown in a heatmap (main figure 5).

Figure S1

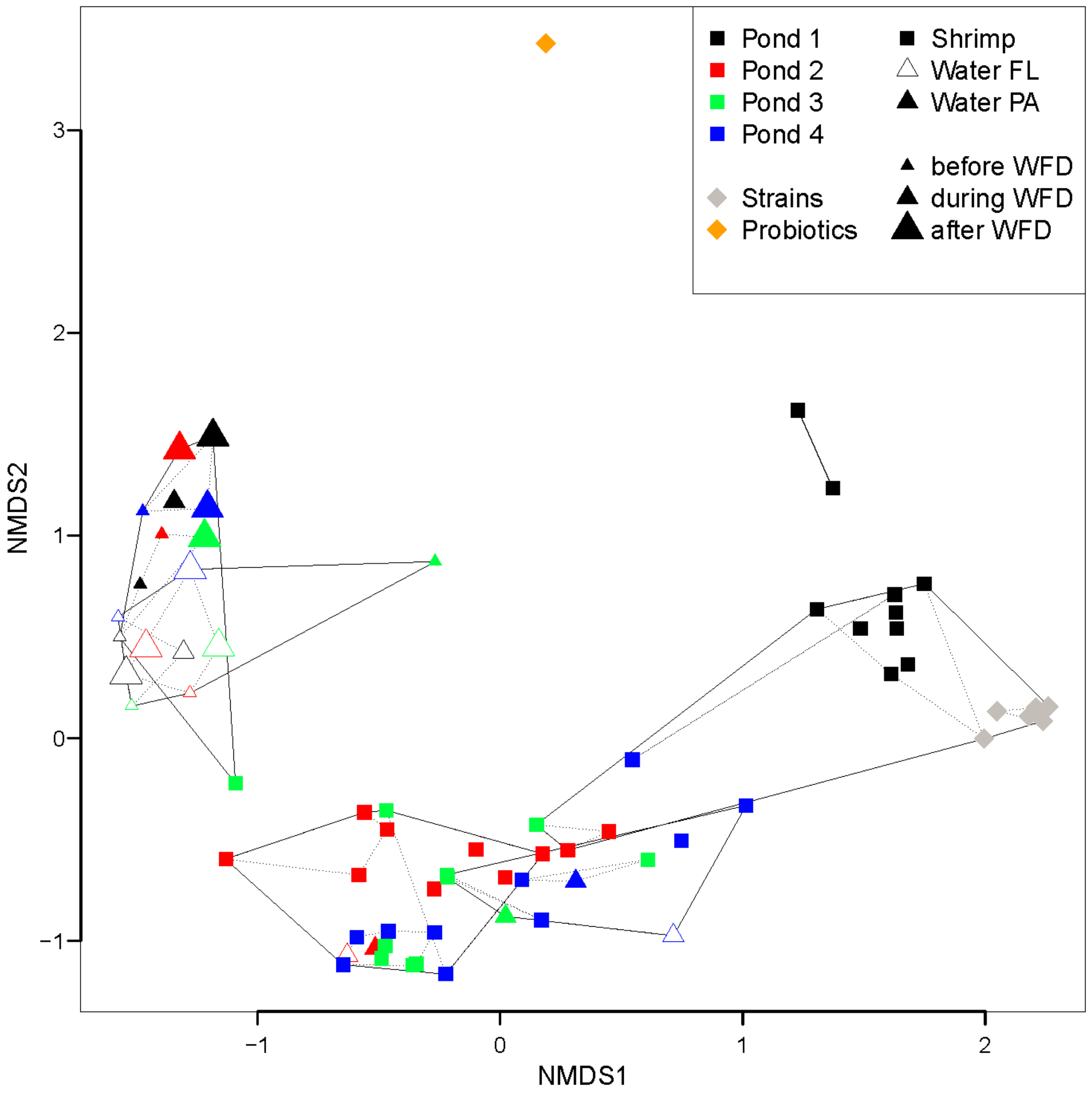

Supplementary Information Figure 1. Non Metric Multidimensional Scaling (NMDS) plot of bacterial community compositions in water samples and shrimps (intestinal bacteria: black squares, faecal bacteria in red, green and blue), bacterial strains (grey diamond) and probiotics (orange diamond). Pond 1: pond with healthy shrimps. Ponds 2, 3 and 4: ponds with diseased shrimps.
